# Temporal Variations in Physico-Chemical and Microbiological Characteristics of Mvudi River, South Africa

**DOI:** 10.3390/ijerph120404128

**Published:** 2015-04-14

**Authors:** Joshua N. Edokpayi, John O. Odiyo, Titus A.M. Msagati, Natasha Potgieter

**Affiliations:** 1 Department of Hydrology and Water Resources, University of Venda, Private Bag X5050, Thohoyandou 0950, South Africa; E-Mail: john.odiyo@univen.ac.za; 2 College of Science, Engineering and Technology, Nanotechnology and Water Sustainability Research Unit, Florida Science Campus, University of South Africa, Roodepoort 1709, Johannesburg, South Africa; E-Mail: msagatam@unisa.ac.za; 3 Department of Microbiology, University of Venda, Private Bag X5050, Thohoyandou 0950, South Africa; E-Mail: natasha.potgieter@univen.ac.za

**Keywords:** *E. coli*, *Enterococci*, public health, water quality, wet and dry seasons

## Abstract

Surface water has been a source of domestic water due to shortage of potable water in most rural areas. This study was carried out to evaluate the level of contamination of Mvudi River in South Africa by measuring turbidity, electrical conductivity (EC), pH, concentrations of nitrate, fluoride, chloride, and sulphate. *E. coli* and *Enterococci* were analysed using membrane filtration technique. Average pH, EC and Turbidity values were in the range of 7.2–7.7, 10.5–16.1 mS/m and 1.3–437.5 NTU, respectively. The mean concentrations of fluoride, chloride, nitrate and sulphate for both the wet and the dry seasons were 0.11 mg/L and 0.27 mg/L, 9.35 mg/L and 14.82 mg/L, 3.25 mg/L and 6.87 mg/L, 3.24 mg/L and 0.70 mg/L, respectively. *E. coli* and *Enterococci* counts for both the wet and the dry seasons were 4.81 × 10^3^ (log = 3.68) and 5.22 × 10^3^ (log = 3.72), 3.4 × 10^3^ (log = 3.53) and 1.22 × 10^3^ (log = 3.09), per 100 mL of water, respectively. The count of *E. coli* for both seasons did not vary significantly (*p* > 0.05) but *Enterococci* count varied significantly (*p* < 0.001). All the physico-chemical parameters obtained were within the recommended guidelines of the Department of Water Affairs and Forestry of South Africa and the World Health Organization for domestic and recreational water use for both seasons except turbidity and nitrates. The microbiological parameters exceeded the established guidelines. Mvudi River is contaminated with faecal organisms and should not be used for domestic purposes without proper treatment so as to mitigate the threat it poses to public health.

## 1. Introduction

Water is necessary for healthy living and must be available to consumers in sufficient quantity and at high quality [1]. In developing countries of the world, most rural communities depend on water from wells, ponds, springs and rivers for their domestic needs [2,3]. This is largely due to either the lack of access to or inability to afford the price of potable water [4]. About 1.1 billion people in the world lack basic access to clean, safe and adequate water resources and 85% of them live in rural areas [5]. According to Schaefer [6], 80% of illnesses and deaths in developing countries are due to the use of water of poor quality. 

The Council for Scientific and Industrial Research (CSIR) reported that almost 2.11 million people in South Africa lack access to any safe water infrastructure [7]. Population growth coupled with increased industrialisation, livestock farming and urbanisation have led to frequent contamination of river systems. This is further exacerbated by the lack of adequate sanitation facilities in rural areas resulting in faecal contamination of surface water with its attendant negative effects on human health and the environment [8,9]. Contamination of river systems by point and non-point sources of pollution degrades water quality and affects its use for domestic, agricultural, recreational and aesthetic purposes [10]. Microbial contamination of water is a major threat to public health as water associated diseases comprise about 9.1% of global disease burden [11,12]. Contaminated water endangers both the physical and social wellbeing of all people and it is an offense to human dignity [13]. Drinking and use of faecally contaminated water has far reaching negative health effects such as cholera, diarrhoea, ring worm infestation and schitosomiasis, among others [5,14]. 

CSIR [7] reported that diarrhoea is the third South African’s biggest killer disease, which is not only restricted to children and immune compromised individuals but also among adults between the ages of 45–65 years. A record of an annual death of 1.6 million people is caused by the use of unsafe water and lack of access to basic sanitation and 90% of the victims are children under the age of 5 from developing countries [5]. Most river systems in urban areas are monitored constantly for their microbiological quality but there are limited data on the microbiological quality of rivers in rural communities, which are more prone to contamination. This study was carried out to assess the physico-chemical and microbiological quality of Mvudi River, which is used by residents surrounding it for domestic, recreational and agricultural purposes.

## 2. Experimental Section 

### 2.1. Study Area

**Figure 1:**
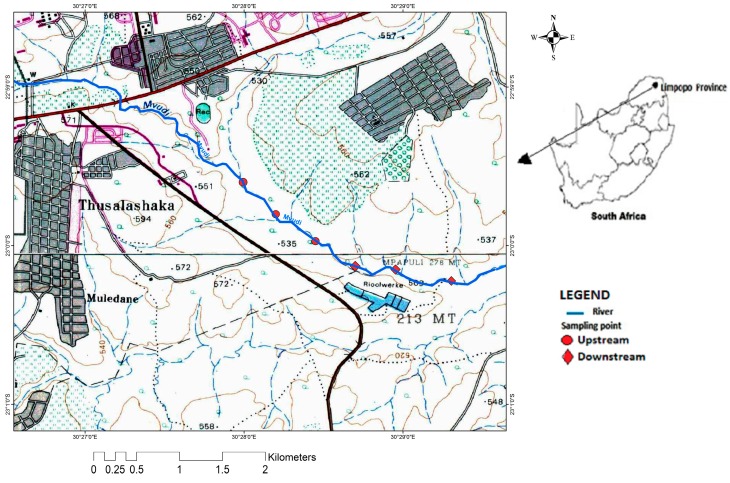
Map of the study area.

Figure 1 shows the study area which is located on geographical coordinates E030°27ʹ59.3″ and E030°28ʹ46.0″ latitude and S22°59ʹ37.5″ and S23°00ʹ10.4″ longitude and falls within the summer climatic condition of South Africa with an average rainfall in the range of 400 to 800 mm annually. Major land uses include informal and formal settlements, subsistence and commercial agriculture, waste disposal sites and wastewater treatment plant. This region is characterized by a warm wet season which is associated with high temperatures up to 40 °C usually between October and March, with peak precipitation in January and February. The cool dry season has a temperature range between 12 °C and 22 °C and begins from April to September [15].

### 2.2. Sampling

Water samples were randomly collected from six points along the Mvudi River using the Direct Sampling method. The sampling was done from January to June 2014. Polypropylene sampling bottles were used for sample collection; they were rinsed with the samples prior to collection from the river. Field measurements of pH and electrical conductivity (EC) were performed using a pH and EC multimeter and turbidity was measured with a turbidimeter. The instruments were calibrated according to the manufacturer guidelines. The samples were transported on ice-chest in a cooler box to Microbiology Laboratory of the University of Venda. The samples for microbial analysis were analysed within 6 h of collection except January samples, which were analysed within 16 h of collection.

### 2.3. Analysis of Microbiological Parameters

*E*. *coli* and *Enterococci* counts were analysed in each sample with the protocol proposed by the American Public Health Agency [16]. Briefly, sample aliquots of 100 mL were filtered using a 0.45 µm pore size, 47 mm diameter Millipore filter membrane. *E.coli* and *Enterococci* bacteria were enumerated at 37 °C on mFC (Acumedia, Pretoria, South Africa) and mEnterococcus agar (Acumedia) plates after 24 and 48 h incubation, respectively. The samples were analysed in duplicate and recorded as colony forming unit per 100 mL.

### 2.4. Anion Analysis

The samples were filtered through 0.45 micron syringe filter and placed in an autosampler connected to an 850 IC professional Ion Chromatograph (Metrohm (pty) Ltd, Pretoria, South Africa). Calibration standards were prepared from multi-element standards of fluoride, chloride, nitrate and sulphate. 1 mg/L, 5 mg/L, 10 mg/L and 20 mg/L were prepared by serial dilution from a stock solution of 100 mg/L. The eluent used was a combination of sodium carbonate (Na_2_CO_3_) and sodium bicarbonate (NaHCO_3_); prepared by weighing accurately 0.1680 g and 0.6784 g into 2L volumetric flask and filled to the mark using ultrapure water (18.2 MΩ). The eluent was degassed before introduction to the IC system. A sample loop of 20 µL, 50 mmol/L of sulfuric acid as the suppressor solution flowing at a rate of 0.5 mL/min was employed. The IC has a flow rate of 0.7 mL/min, maximum and minimum pressure of 15.0 mPa and 0.1 mPa, respectively. The samples were analysed in triplicate.

### 2.5. Validation of Analytical Methodology

In order to validate the analytical methodology, recovery studies were performed. Known concentrations of the test analyte were added to the sample. The concentrations of both the spiked and unspiked samples were determined and percentage recovery was obtained.

### 2.6. Statistical Analyses

Data analyses were performed using Microsoft Excel and SPSS 20.0 statistical software.

## 3. Results and Discussion

### 3.1. Physico-chemical Parameters

The pH values of the river over the study period were in the range of 6.7–8.0. Table 1 shows the average pH values for each of the sampling month. The average pH values of the dry season (April−June) (7.5) was slightly higher than that obtained for the wet season (January−March) (7.4) and fell within the recommended limit of 6–9 set by the Department of Water Affairs and Forestry of South Africa (DWAF) [17] and the World Health organization (WHO) [18] for domestic, recreational and agricultural water use. There is no significant difference in the means of the pH values observed for both the wet and the dry seasons (*p* > 0.05).

**Table 1:** Average levels of physico-chemical parameters in Mvudi River.

Months	pH	Turbidity (NTU)	EC (mS/m)
January	7.4 ± 0.04	429 ± 31	10.5 ± 0.08
February	7.3 ± 0.04	20.4 ± 4.3	15.9 ± 0.66
March	7.7 ± 0.16	17.6 ± 8.2	13.6 ± 1.8
April	7.2 ± 0.22	8.0 ± 0.67	12.8 ± 0.62
May	7.6 ± 0.05	7.8 ± 3.5	16.1 ± 2.13
June	7.6 ± 0.05	1.9 ± 1.16	13.8 ± 1.67
DWAF guidelines	6–9	<0.1	<70
WHO guidelines	6.5–9.5	<0.1	600

pH plays an important role in the speciation and bioavailability of metals in aquatic environment. A pH < 4 will increase the toxicity and bioavailability of most heavy metals while a pH > 9 will influence the toxicity of ammonium ion. The pH values obtained in this study in the dry and the wet seasons are comparable to those reported by Singh *et al.* [19]. Wilbers *et al.* [20] reported a slightly lower pH in the wet season than in the dry season in their studies of spatial and temporal variability of surface water pollution in the Mekong Delta, Vietnam.

Electrical conductivity of rivers is a function of the geology of the area. The EC trend in rivers that flow predominantly across granite rocks which contain no ionisable metals will be generally lower than those that flow across rocks that contain ionisable metals which are readily leached into the river [21]. From Table 1, the EC values obtained were different in each sampling month. The lowest EC value was obtained in January and the highest in May. This result was unexpected as the wet season which is characterized with heavy rainfall is supposed to be accompanied with high EC values due to high surface runoff from agricultural lands and roads as reported by Shabalala *et al.* [21] and Anhwange *et al.* [22]. 

A similar result to the findings from this study was reported by Vaishali *et al.* [23]. The EC determined is in the same range in both seasons and statistical analysis of the data obtained for both seasons showed no significant difference (*p* > 0.05). The value of EC usually gives an indication of the presence of dissolved ions in water [24] and the presence of these ions can alter the taste of water and also contribute to the hardness of water. It also gives an indication of the total dissolved solids present in the water. Water with high EC values is not suitable for domestic purposes and irrigation as it can lead to the salinity of agricultural soils. The obtained values were within the guideline value of DWAF (70 mS/m) [17] and WHO (600 mS/m) [18].

The turbidity values obtained decreased as expected from January to June. The values were in the range of 13.3–473 NTU in the wet season and 1.3–14.7 NTU in the dry season. The high turbidity values obtained during the wet season can be attributed to high incidences of rainfall, which lead to increased erosion and surface runoff carrying a lot of suspended materials into the river. High turbidity values indicate the possible presence of micro-organisms, clays, silts and other suspended solids in water, which affect its aesthetic value by causing it to appear cloudy [25].

Turbidity is usually associated with reduced penetration of light rays which can adversely affect benthic organisms. The low values of turbidity in the dry season could be due to reduced circulation velocity of water, causing the suspended particles to settle down in sediment [22]. The turbidity values obtained in both seasons exceeded the DWAF and WHO guidelines of <1 NTU for domestic water use [17]. Statistical treatment of the data showed a significant difference (*p* < 0.006) for both the wet and the dry seasons. Seasonal variation in turbidity levels were recorded by Lin *et al.* [26] and Fatoki *et al.* [27] on their investigation of Umgeni and Umtaka Rivers in KwaZulu Natal and Eastern Cape Provinces of South Africa, respectively. 

### 3.2. Anions

The validation test performed on the analytical methods employed gave reproducible results with acceptable recoveries. Recovery percentages were 92.5% for fluoride, 95.7% for chloride, 95.1% for nitrate and 97.3% for sulphate. The results obtained are presented in Table 2. The highest fluoride (F^−^) concentration (0.150 ± 0.05 mg/L) was obtained in March and the lowest in January (0.058 mg/L) (Table 2). The mean concentration of F^−^ for the wet and the dry seasons were 0.11 mg/L and 0.27 mg/L, respectively. The low F^−^ concentration in the wet season could be due to dilution as a result of heavy rains. *p*-Value of 0.428 was obtained which shows that the mean difference for both wet and the dry seasons did not vary significantly. The concentrations obtained for both seasons were within the limit of 1 mg/L and 1.5 mg/L set by DWAF [17] and WHO [18] for domestic water use. This result is comparable to those obtained by Lalaury and Gopmath [28]. Ravindra *et al.* [29] reported a slightly higher concentration of fluoride in the wet season than in the dry season. Low F^−^ concentration is beneficial to man as it aids the calcification of dental enamel, but when the concentration exceeds 1 mg/L, it can cause dental and skeletal fluorosis which can be severe at higher concentrations [28].

**Table 2:** Average monthly concentrations of anions in Mvudi River.

Anions (mg/L)	Sampling Months					
	January	February	March	April	May	June
Fluoride	0.058 ± 0.01	0.106 ± 0.01	0.15 ± 0.05	0.064 ± 0.04	0.105 ± 0.06	0.105 ± 0.06
Chloride	5.59 ± 0.31	8.90 ± 1.27	13.56 ± 9.53	9.27 ± 0.14	15.75 ± 6.21	19.48 ± 12.0
Nitrate	5.25 ± 4.72	1.97 ± 0.52	2.53 ± 1.37	4.86 ± 3.88	7.75 ± 5.86	8.17 ± 5.52
Sulphate	2.79 ± 2.1	1.89 ± 0.63	5.04 ± 3.36	0.66 ± 0.07	0.74 ± 0.15	0.76 ± 0.15

The average concentration of chloride (Cl^−^) increased from 5.59 ± 0.31 mg/L in January to 8.90 ± 1.27 mg/L in February and to 13.56 ± 9.53 mg/L in March; a decrease to 9.27 ± 0.14 mg/L was observed in April but an increase was observed again in May (15.72 ± 6.21 mg/L) and June (19.48 ± 12.08 mg/L). The Cl^−^ concentrations were higher in the dry season than in the wet season. This could possibly be due to high dilution of chloride as a result of high rainfall in the wet season. The difference in the means for the wet and the dry seasons was statistically different (*p* < 0.015). The concentrations obtained fell within the set guidelines of DWAF (100 mg/L) [17] and WHO (250 mg/L) [18]. These low concentrations agree with the low level of electrical conductivity observed for both seasons.

The average nitrate concentration varied between 4.68 ± 3.88 mg/L, 7.75 ± 5.86 mg/L and 8.17 ± 5.22 mg/L in the months of April, May and June, respectively; while 5.25 ± 4.72 mg/L, 1.97 ± 0.52 mg/L and 2.53 ± 1.37 mg/L values were recorded for January, February and March, respectively. The average nitrate (NO_3_^−^) concentration was higher in the dry season than in the wet season. A similar observation was reported by Anhwange *et al.* [22], Adeyemo *et al.* [30] and Shrestha and Kazava [31]. The concentrations of NO_3_^−^ are usually built up during the dry season while in the wet season there is high dilution due to high rainfall events. These are the possible reasons for the slightly higher concentration of nitrate observed in the dry season [30].

The concentrations of NO_3_^−^ did not exceed the DWAF and WHO threshold limits for domestic water use but the concentration determined can induce eutrouphication. Nitrate concentration in the range of 2.5–10 mg/L can render a water body eutrouphic and consequently lead to the blossoming of algae, some of which are poisonous [17,21]. A significant difference in the mean values of the concentrations was observed (*p* < 0.035) for the wet and the dry seasons. High nitrate concentrations in water usually lead to eutrouphication with its consequences and nitrate concentration exceeding 23 mg/L has been linked to methaemoglobinaemia condition in children [17].

Very low concentrations of sulphate (SO_4_^2−^) were observed during the sampling months. The highest SO_4_^2−^ concentrations were observed in March (5.04 ± 3.36 mg/L), January (2.79 ± 2.1 mg/L) and February (1.89 ± 0.63 mg/L) all in the wet season as shown in Table 2. This could be attributed to high surface runoffs from roads carrying sulphate minerals into the river. Ravindra *et al.* [29] in their studies of seasonal variations in physico-chemical characteristics of River Yamuna reported a higher sulphate concentration in the wet season than in the dry season. The concentrations in the dry season were in the range 0.66–0.74 mg/L. The SO_4_^2−^ concentrations for both seasons were within the guidelines of DWAF [17] and WHO for domestic water use [18] of 200 mg/L and 250 mg/L, respectively. The mean difference for both seasons varied significantly (*p* < 0.005). 

Correlation studies were performed on the concentrations of the anions during the sampling period. Fluoride showed a significant correlation with chloride (r = 0.382, *p* < 0.05), nitrate also showed a positive and significant correlation with chloride (r = 0.457, *p* < 0.01). Sulphate did not correlate significantly with any of the other anions. Table 3 shows the average concentrations of the anions upstream and downstream of the river. Generally, higher concentrations were observed downstream than upstream of the river. This could be attributed to various land use activities such as agricultural, small scale industries and wastewater treatment works located along the river from upstream to downstream which can result to higher concentrations of pollutants downstream [32].

**Table 3:** Average anion concentrations upstream (Us) and downstream (Ds) of Mvudi River.

Anions (mg/L)	Sampling Months					
	January	February	March	April	May	June
Fluoride Us	0.052 ± 0.01	0.098 ± 0.01	0.131 ± 0.0	0.056 ± 0.04	0.068 ± 0.09	0.069 ± 0.08
Ds	0.06 ± 0.01	0.110 ± 0.01	0.159 ± 0.06	0.068 ± 0.04	0.130 ± 0.06	0.113 ± 0.05
Chloride Us	5.32 ± 0.29	7.33 ± 0.18	9.93 ± 2.97	9.15 ± 0.66	12.13 ± 3.15	12.79 ± 2.95
Ds	5.73 ± 0.23	9.68 ± 0.64	15.37 ± 11.64	9.33 ± 0.12	12.31 ± 6.95	22.79 ± 13.98
Nitrate Us	4.75 ± 5.92	1.43 ± 0.01	3.07 ± 1.39	2.06 ± 0.85	9.36 ± 11.46	5.21 ± 1.53
Ds	7.03 ± 6.94	2.38 ± 0.40	3.01 ± 1.49	6.95 ± 4.24	9.16 ± 3.30	12.56 ± 6.43
Sulphate Us	1.52 ± 0.01	1.14 ± 0.33	2.29 ± 0.30	0.641 ± 0.05	0.695 ± 0.13	0.834 ± 0.21
Ds	3.35 ± 2.49	2.26 ± 0.27	6.29 ± 3.36	1.34 ± 0.09	0.767 ± 0.16	0.654 ± 0.09

### 3.3. Microbiological Parameters

**Figure 2:**
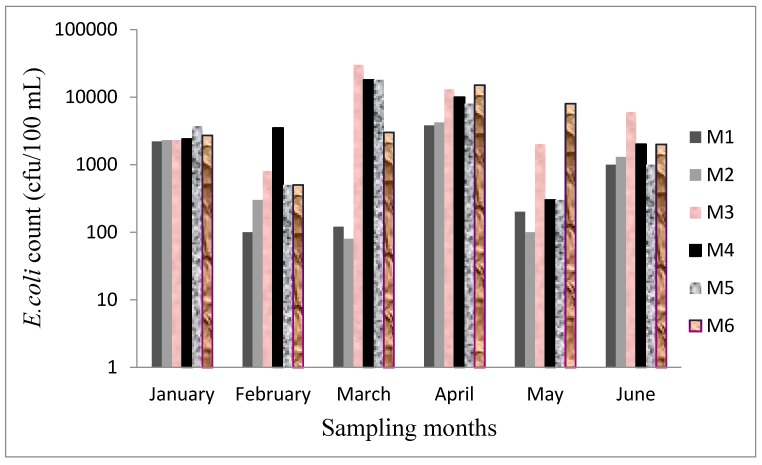
*E.coli* count in each sampling site of Mvudi River.

**Figure 3:**
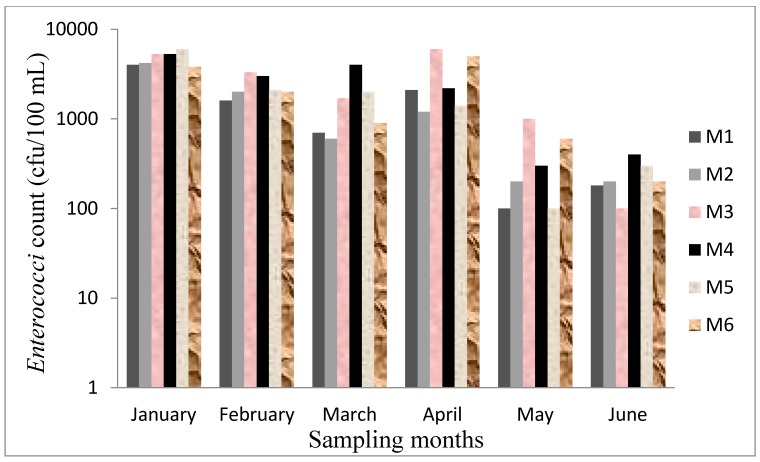
*Enterococci* count in each sampling site of Mvudi River.

Faecal contamination is of global concern owing to the negative health risks associated with it. Figure 2 and Figure 3 show the average counts of *E. coli* and *Enterococci* observed in this study. The highest count of *E. coli* was observed in March in the wet season (log = 4.06) followed by April in the dry season (log = 3.95). The mean count of *E. coli* recorded for the wet and the dry seasons were 4.81 × 10^3^ (log = 3.86) and 5.22 × 10^3^ (log = 3.72), respectively. The average count of *E. coli* was slightly higher in the dry season when compared with the wet season. These levels exceeded the DWAF and WHO recommended guidelines for domestic and recreational use of water [17,18].

Abstraction of water from Mvudi River is thus regarded a risk to the health of people that use the water for various purposes. The counts obtained were not statistically different (*p* > 0.05) for both seasons.

**Figure 4:**
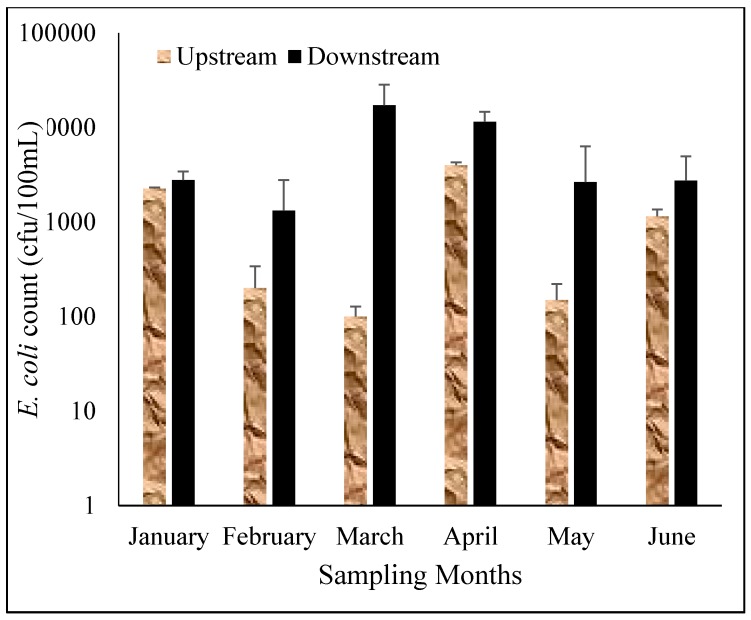
*E. coli* count in the upstream and downstream of Mvudi River.

*Enterococci* count varied differently in each of the sampling months; it decreased from 5.23 × 10^3^ (log = 3.73) in January to 3.33 × 10^3^ (log = 3.52) in February and a further decrease was observed in March (log = 322). An increase was observed in April (log = 3.47) and a sharp decrease was again recorded in May (log = 2.58) and June (log = 2.48). The levels obtained were higher in the wet season than in the dry season. Differences in the means for both seasons were significantly different (*p* < 0.001). The downstream of the river recorded higher counts of *E. coli* and *Enterococci* as shown in Figure 3 and Figure 4 than the upstream. The data for both seasons exceeded the recommended guidelines set by DWAF and WHO for water use [17,18].

**Figure 5:**
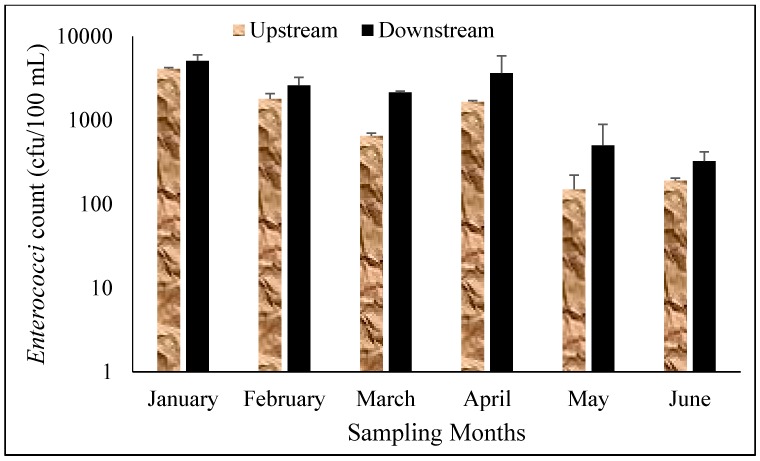
*Enterococci* count in the upstream and downstream of Mvudi River.

Seasonal variations had influence on the levels of faecal bacteria thereby making it very difficult to predict their value at any given time. Bacterial counts are usually expected to be higher during the wet season because of high rainfall events and are more prevalent in turbid water [33] but high ultra violet radiation from sunlight can reduce the bacterial counts in the rainy season which is associated with high sunshine in Limpopo Province of South Africa. *Enterococci* count was higher in the wet season than in the dry season which was opposite to *E. coli* count which was higher in the dry season than in the wet season. The high levels of both indicator organisms found in this study is of a great concern as it can negatively affect the health of the users of this resource. Various seasonal patterns of feacal indicator organisms have been reported for several rivers in South Africa. Chigor *et al.* [34] and Lin *et al.* [25] did not observe any significant differences in the counts of faecal indicator organisms for both the wet and dry seasons on Buffalo and Umgeni Rivers in South Africa. Fatoki *et al.* [27] observed a higher count of faecal indicator organisms in the rainy season than in the dry season while, Sibanda *et al.* [35] reported a higher count of faecal indicator organisms in Dryini sampling point during the dry season than in the wet season in their studies on Tyume River in Eastern Cape Province in South Africa. Figure 4 and Figure 5 show the average counts of *E. coli* and *Enterococc*i on the upstream and downstream of the river. 

Generally, the levels of faecal indicator organisms were higher downstream than upstream of the river and can be attributed to various anthropogenic activities occurring along the river and could also be due to effluents from wastewater treatment works.

## 4. Conclusions

From the experimental observation, the physico-chemical parameters were within the set guidelines of DWAF and WHO for domestic water use except for turbidity. The microbiological parameters exceeded the guideline values of 0 cfu/100 mL, 30 cfu/100 mL, and ≤1000 cfu/100 mL for domestic, recreational and irrigation water use [36,37]. pH, EC and the concentrations of F^−^ and *E. coli* count did not vary significantly for both the wet and the dry seasons. Turbidity values, the concentrations of NO_3_^−^, Cl^−^, SO_4_^2−^, and *Enterococci* count varied significantly for both seasons. Generally, the downstream of the river was more contaminated than the upstream. It is recommended that the water should be properly disinfected before any use. Measures should be put in place to discourage abstraction of water from this river in order to protect the health of the communities that rely on it for domestic, agricultural and recreational purposes. 
